# Grazing Intensity Shapes Vegetation Structure and Soil Characteristics in High‐Elevation Rangelands of Nepal

**DOI:** 10.1002/ece3.72689

**Published:** 2025-12-17

**Authors:** Tulasa Chaudhary, Uttam Babu Shrestha, Uma Dhungel, Nishan Baral, Bharat Babu Shrestha, Shikui Dong

**Affiliations:** ^1^ Global Institute for Interdisciplinary Studies Kathmandu Nepal; ^2^ Central Department of Botany Tribhuvan University Kathmandu Nepal; ^3^ School of Grassland Science Beijing Forestry University Beijing China

**Keywords:** community composition, ecosystem health, intermediate disturbance hypothesis, pastoralism, rangeland productivity, subalpine rangeland

## Abstract

Understanding the ecological impacts of livestock grazing on rangeland conditions is essential for developing sustainable rangeland management strategies that balance livestock production and ecosystem health. However, there is a paucity of field experiments measuring the impacts of grazing on rangeland health in the Himalayan region. This study assessed the impacts of four grazing intensities—non‐grazed (NG), lightly grazed (LG), moderately grazed (MG), and highly grazed (HG)—on plant species diversity, community composition, plant height, biomass production, and soil physicochemical properties across three sub‐alpine rangelands in Jumla district, western Nepal. A total of 113 plant species were recorded, dominated by forbs (87.6%), with fewer graminoids, pteridophytes, and shrubs. Species richness increased along the grazing gradient from HG to NG, with MG plots supporting high diversity and productivity. Non‐metric multidimensional scaling (NMDS) ordination revealed distinct species assemblages under NG, while MG and LG showed overlapping plant communities. Plant height and biomass were the highest in NG plots and declined with increased grazing intensity, with elevation exerting a negative effect on both. Soil organic carbon and nitrogen were generally higher under NG and MG, although responses varied across sites. Our results support the intermediate disturbance hypothesis and highlight the ecological benefits of moderate grazing, although these effects are largely site‐specific. The complex interaction between grazing pressure and environmental conditions underscores the importance of site‐specific and adaptive management strategies to sustain rangeland health. Our findings provide valuable evidence for designing evidence‐based sustainable rangeland management strategies in Nepal and comparable high‐altitude regions.

## Graphical abstract

This study assessed the impacts of four grazing intensities—non‐grazed (NG), lightly grazed (LG), moderately grazed (MG), and highly grazed (HG)—on plant species diversity, community composition, plant height, biomass production, and soil physicochemical properties across three sub‐alpine rangelands in Jumla district, western Nepal. Our results support the intermediate disturbance hypothesis and highlight the ecological benefits of moderate grazing. Our findings provide valuable evidence for designing evidence‐based sustainable rangeland management strategies in Nepal and comparable high‐altitude regions.

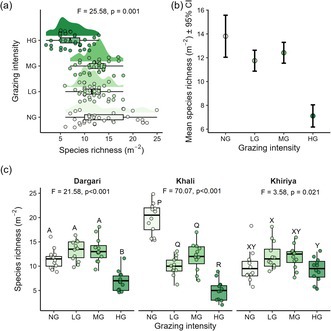

## 1 Introduction

High altitude rangelands in the Himalaya are extensive natural landscapes characterized by low and erratic precipitation, long cold winters, high irradiance, significant seasonal temperature and precipitation fluctuations, and vegetation dominated by native grasses, forbs, shrubs, and scattered trees (Nautiyal et al. 2001; Dong et al. 2009; Ning et al. 2013). Although these landscapes are primarily used for livestock grazing, these rangelands provide a wide range of ecosystem services such as wildlife habitats including globally threatened species, carbon sequestration, water storage and regulation, and the provision of high‐value medicinal species, wild foods and mushrooms, and opportunities for eco‐tourism (Dong et al. 2010; Joshi et al. 2013). These rangelands also support the livelihoods and traditional way of life, including transhumance practices (Aryal et al. 2013). They are vital for both local and migratory pastoral communities who rely on livestock farming and the collection of medicinal species, contributing significantly to household incomes (Hruska et al. 2017; Ingty 2021). In Nepal, the high‐altitude rangelands serve as the primary source of forage for livestock (Dong et al. 2007), supplying up to 98% of total feed requirements in the high mountain regions (Dhakal et al. 2019). Thus, high‐altitude rangelands form the ecological and economic backbone of mountain livelihoods, sustaining biodiversity, culture, and pastoral production systems.

Livestock grazing in rangeland ecosystems plays a critical role in shaping their structure, functions, and the ecosystem services they provide. However, high grazing pressure may act as a “disturbance,” negatively affecting both biotic and abiotic factors, leading to degradation of rangelands (Zaady et al. 2001). On the global scale, livestock grazing has been shown to reduce primary production by 26%, water conservation by 18%, and carbon sequestration by 19%, while showing weaker negative effects on nutrient cycling (−11%) and decomposition (−12%) (Niu et al. 2024). However, when appropriately managed, grazing can also serve as a beneficial ecological tool, maintaining vegetation dynamics, supporting soil nutrient cycling, and regulating overall ecosystem processes and functions (Alkemade et al. 2013).

Studies report varied effects of grazing intensity on vegetation structure, productivity, and soil properties. For example, livestock exclusion for 2 years negatively affected plant richness but did not affect insect community composition (Ubach et al. 2023). Areas under grazing bans exhibited lower plant diversity compared to traditionally grazed areas (Ingty 2021). Similarly, complete grazing exclusion for longer periods may lead to secondary succession and the formation of complex plant community structures that include greater herbaceous biomass and shrub encroachment, ultimately resulting in the loss of native rangeland biodiversity (Oba et al. 2001). However, controlled grazing can improve forage production and small ruminant productivity (Islam et al. 2018). Controlled grazing increased species diversity, species richness, including native species richness, and productivity (Oba et al. 2001; Huston 2004; Thapa et al. 2016; Lu et al. 2017; Getabalew and Alemneh 2019). Moderately grazed forests had higher tree richness, diversity, seedling density, and natural regeneration than low or heavily grazed forests at the tree line ecotone (Basnet et al. 2024). Plots located at moderate to far distances from animal sheds (*goths*) showed higher overall species richness, including rare species (Aryal et al. 2015). On the other hand, overgrazing can severely degrade rangeland conditions by reducing biomass, lowering vegetation cover of desirable species, increasing weed and unpalatable or grazing‐tolerant species, and causing soil compaction due to trampling and destruction of the root system (Reid et al. 2010; Getabalew and Alemneh 2019). So, heavy grazing was found responsible for both reduced diversity and aboveground net primary productivity with increased belowground productivity (Zhang, Delgado‐Baquerizo, et al. 2023) as intense grazing can reduce vegetation cover resulting in bare ground (Ostoja et al. 2014). Overall, these patterns of how varying grazing intensities influence rangeland biodiversity clearly support the intermediate disturbance hypothesis (IDH), which emphasizes that both too little and too much disturbance can negatively affect species diversity within a given area (Connell 1978; Aronson and Precht 1995).

In addition, grazing also affects the soil and water cycle of the rangeland ecosystems, affecting their soil and water conservation functions. Grazing can induce an increase in soil moisture, depending on the habitat (Zaady et al. 2001). Trampling by livestock can lead to soil compaction, altering hydrology by reducing water infiltration (Greenwood and McKenzie 2001). Livestock trampling routes can transform the vegetation mosaic, increase complexity, strengthen the spatial redistribution of water and soil resources at the patch scale, and decrease hydrological connectivity (Stavi et al. 2021). Over‐grazing can decrease water infiltration and increase soil erosion, driving habitat degradation (Eldridge and Delgado‐Baquerizo 2017; Lu et al. 2017). Grazing animals influence nutrient cycling primarily through the redistribution of nutrients via dung and urine, often concentrating nutrients in favored grazing or resting areas (Barzan et al. 2021). Deposition of dung and urine by livestock can increase soil organic matter and nutrient content as well (Ingty 2021; Basnet et al. 2024).

Despite its positive and negative effects, grazing is generally perceived as detrimental to ecosystem health by forest officials in Nepal and is often cited as a major cause of environmental degradation (DFRS 2015; Acharya and Baral 2017). Hence, after the implementation of Nepal's community forestry program, grazing was banned in the rangelands of many areas (Banjade and Paudel 2008; Acharya and Baral 2017). In contrast, herders evaluate rangeland quality primarily through the lens of livestock productivity, emphasizing factors such as forage quality, palatability, and the capacity to support high pastoral production, with the goal of maximizing returns (Inam‐ur‐Rahim and Maselli 2004; Kong et al. 2015). In this context, understanding how grazing intensity influences key ecological aspects of rangelands is essential for developing sustainable management strategies that balance livestock production with biodiversity conservation goals. Furthermore, it is important to recognize that rangeland productivity is shaped not solely by grazing pressure but also by site‐specific environmental conditions, including climate, topography, vegetation type, soil characteristics, land use history, and land management practices (Zaady et al. 2001; Wang et al. 2012; Alkemade et al. 2013; Li et al. 2018; Ahmed et al. 2020; Niu et al. 2024). Thus, quantifying grazing intensity and its ecological consequences by accounting for multiple site‐specific variables is essential for effective rangeland management.

This study aimed to assess site‐specific grazing intensity and its impacts on plant diversity, biomass production, plant height, and soil physicochemical properties in three rangelands within Jumla district, western Nepal, where rangelands are vital for pastoral livelihoods and serve as key areas for livestock grazing and medicinal plant collection. We tested whether grazing is a major factor affecting rangeland conditions and whether moderate grazing positively influences overall rangeland health. The findings will help develop site‐specific, data‐informed management strategies that promote both ecosystem health and livestock productivity.

## 2 Materials and Methods

### 2.1 Study Area

**FIGURE 1:**
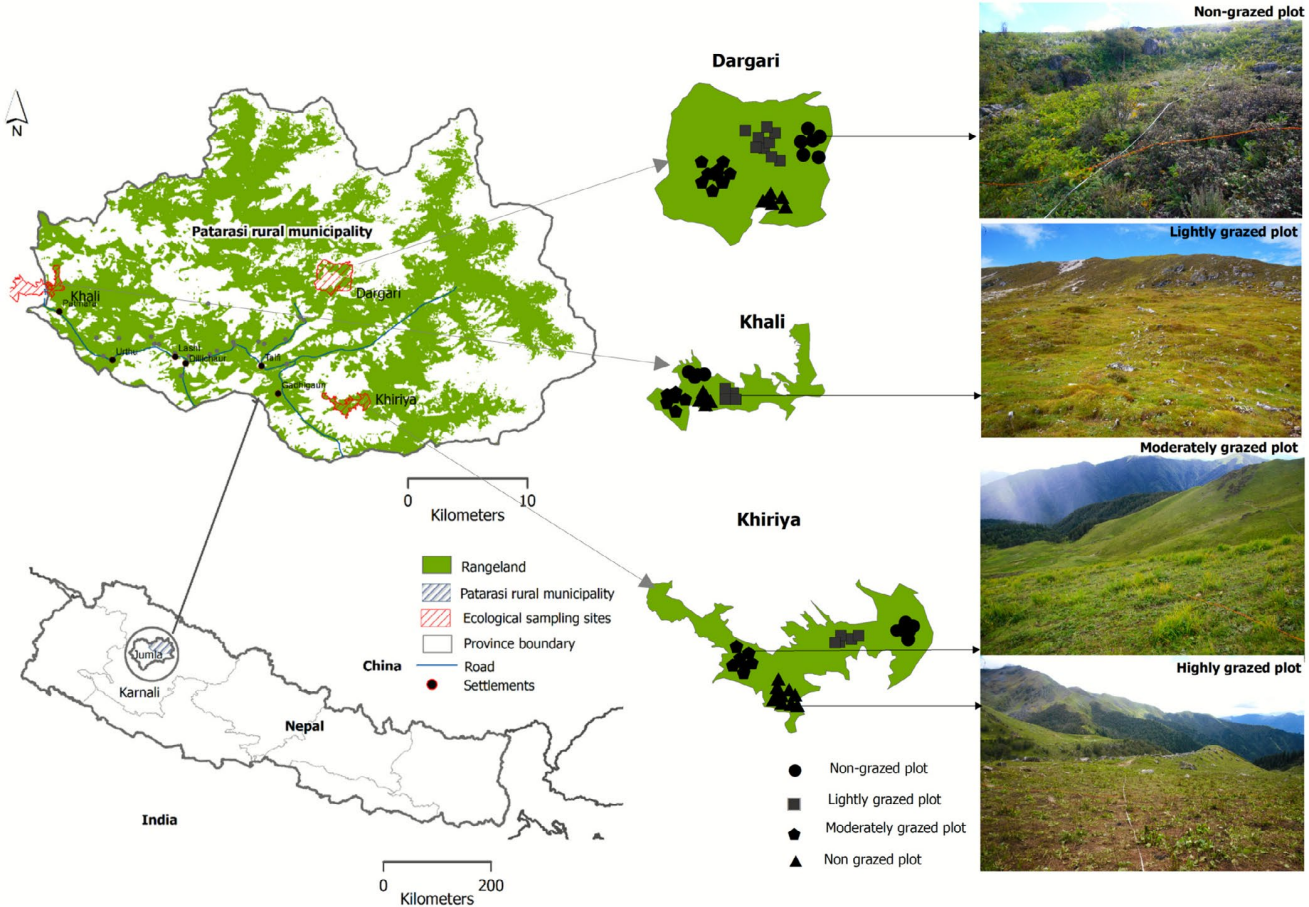
Map of Patarasi Rural Municipality showing the locations of three surveyed rangelands and photographs of plots across the four grazing intensities (Black markers denoted a sampling plot; the smaller number in map is because of overlap).

This study was conducted in grazing rangelands located within Patarasi Rural Municipality (RM), Jumla district, Karnali province in western Nepal (Figure 1). Based on daily climate data from 1951 to 2024, the average annual temperature in the study area is −1.72°C ± 0.74°C and average annual precipitation is approximately 1372 mm (Global Institute for Interdisciplinary Studies (GIIS) 2024). Patarasi Rural Municipality encompasses around 44,192 ha of rangelands, contributing 54% of the total area of the municipality (Karra et al. 2021, Global Institute for Interdisciplinary Studies (GIIS) 2024).

Ecological surveys were carried out in September 2024 in three high‐altitude rangelands: Khiriya (29.253419° N, 82.376948° E; elevation: 3940 m a.s.l.; area: 246 ha), Dargari (29.365937° N, 82.392548° E; elevation: 4136 m a.s.l.; area: 588 ha), and Khali (29.358636° N, 82.159057° E; elevation: 3532 m a.s.l.; area: 417 ha). All the studied rangelands serve mainly as summer grazing sites for different settlements of Patarasi RM and neighboring areas. In Khiriya rangeland, around 300 households from Ward 1 of Patarasi RM reside as a winter rangeland for yak herders from Chhota village in Dolpa district. Dargari rangeland, managed by the Furkesalla Community Forest User Group (CFUG), is used for 1 month during summer by herders from over 300 households in Ward 2. Khali rangeland, managed by the Maharudra CFUG, spans the borders of Patarasi RM, Kanakasundari RM, and Chandannath Municipality in Jumla district. It supports grazing during the spring and monsoon seasons for about 276 households from Ward 7. Livestock grazed in these rangelands include sheep, goats, cattle, horses, mules, and jhopa (a hybrid of cow and yak), while sheep and goats are the dominant livestock grazing at all three selected rangeland sites during the summer. In addition to grazing, all three sites are important for the collection of medicinal plants, contributing significantly to herders' livelihoods.

### 2.2 Experimental Design

#### 2.2.1 Experimental Plot Set Up

To study the impact of livestock grazing on rangeland vegetation and soil properties, a site of at least 100 ha (1 km^2^) was selected at each of the three rangelands, ensuring the presence of four distinct grazing gradients. Several factors influence grazing intensity, including access to water, distance from herders' sheds (goths), and slope of the terrain, as steep slopes are generally unsuitable for grazing. Thus, a single area may experience varying levels of grazing pressure, as livestock tend to graze based on water availability, with areas closer to herders' sheds naturally experiencing higher grazing pressure. However, direct measurement of grazing intensity is complex due to the lack of standardized quantitative indicators (Wang et al. 2017). Therefore, the grazing gradients were identified by qualitative description by using the piosphere concept through a combination of visual assessment and consultations with local herders, following criteria outlined by Alkemade et al. (2013) and Wang et al. (2017). For this, soil indicators included visible signs of compaction and surface exposure, while vegetation indicators involved decreased cover of palatable species and increased presence of unpalatable, shrubby species. Moreover, signs of overgrazing, such as heavy dung deposition and levels of trampling, were also recorded (Table 1). However, it was very difficult to identify completely ungrazed areas, as no physical fencing existed, and these natural grasslands have historically served as common resource pools for local communities. Therefore, after consulting with local herders, we selected the farthest sites, with steep terrain, where grazing rarely occurs, as non‐grazed areas. After the selection of sites, four circular subplots (30 m in diameter) were established within each site to represent the four grazing classes: non‐grazed (NG), lightly grazed (LG), moderately grazed (MG), and highly grazed (HG) rangelands. These grazing intensity plots were spatially independent and arranged following a randomized block design.

**TABLE 1:** Definition of grazing intensity classes and their indicators (adopted from Wang et al. 2017, with modifications).

Grazing intensity	Definition	Indicators				
		Mean distance to herder's shed (m, 95% confidence interval)	Mean distance to water source (m, 95% confidence interval)	Degree of trampling (0–3, visual estimation)	Mean number of plots with dung	Mean bare ground cover (%, 95% confidence interval)
Highly grazed (HG)	Plots located to herders' sheds where livestock graze within a limited area and bare ground cover exceeds vegetation cover	132 (23.3, 241)	218 (80.9, 356)	3	12	73.3 (54.4, 92.3)
Moderately grazed (MG)	Plots located at intermediate distances between HG and LG sites	497 (164, 1158)	315 (34.3, 595)	2	9	38.3 (31.2, 45.5)
Lightly grazed (LG)	Plots located farther from herders' sheds where livestock graze over a larger area and has lower grazing pressure	581 (23.9, 1137)	463 (195, 731)	1	6	17 (10.4, 23.6)
Non grazed (NG)	Farthest from herders' sheds and water source and dominated by shrubs and bushes	759 (335, 1183)	561 (358, 1481)	0	1	8.33 (1.16, 15.5)

**FIGURE 2:**
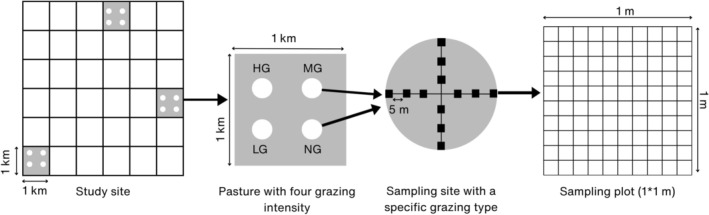
A schematic of sampling methods (HG, highly grazed; LG, lightly grazed; MG, moderately grazed; NG, non‐grazed).

Each 30‐m circular plot was subdivided by drawing two perpendicular transects: one along the north–south (N–S) axis and another along the east–west (E–W) axis, intersecting at the center perpendicularly (Figure 2). Along each transect, six 1 m × 1 m quadrats were placed at 5‐m intervals, starting at one edge and extending across the plot. This resulted in a total of 12 quadrats within each 30‐m circular subplot (i.e., each grazing intensity). Altogether, 144 quadrats were sampled across the three rangelands (12 quadrats per subplot × 4 grazing gradients × 3 study sites). For each subplot, geographic coordinates (latitude, longitude) and elevation were recorded.

#### 2.2.2 Sample Collection and Laboratory Analysis

##### 2.2.2.1 Measurement of Plant Cover and Height

In each quadrat, all plant species present were recorded along with their estimated cover and height. Plant cover was visually estimated, using the Daubenmire Cover‐class Method, which categorizes the percentage of ground covered by each species into one of six cover classes: 1 = 0%–5%, 2 = 5%–25%, 3 = 25%–50%, 4 = 50%–75%, 5 = 75%–95%, and 6 = 95%–100% (Daubenmire 1959). Plant heights were measured by selecting the 10 tallest individuals per species within each quadrat. If fewer than 10 individuals were present, all individuals were measured. Voucher specimens of each recorded species were collected and later identified at the National Herbarium and Plant Laboratories (KATH), Lalitpur, Nepal. The identified species were further categorized into four lifeforms, including forbs, graminoids (grasses and sedges), shrubs and pteridophytes. All herbaceous annuals and perennials, excluding grasses and sedges were grouped under forbs, while ferns were categorized separately as pteridophytes.

##### 2.2.2.2 Plant Biomass Collection and Estimation

For biomass estimation, four quadrats with visibly highest biomass were selected from the 12 quadrats in each 30‐m subplot. This resulted in 48 biomass samples in total (4 quadrats × 4 grazing gradients × 3 sites). Aboveground plant materials were clipped at the surface of the soil using scissors. Visible litter was also collected and separated into either grass or forb categories. Collected samples were first air‐dried in the shade and then oven‐dried at 70°C for 72 h (Perez‐Harguindeguy et al. 2013). Dried biomass was weighed using a digital balance to determine aboveground biomass.

##### 2.2.2.3 Soil Chemical Analysis

Soil samples were collected from four selected quadrats (out of 12 quadrats per subplot), with approximately 100 g taken from a depth of 10–15 cm in each quadrat. Samples were air‐dried and sieved through a 425‐μm mesh before laboratory analysis. Soil pH was measured using an electrometric method in a 1:2 soil‐to‐distilled water suspension with a digital pH meter (EI Instrument, Model: Alpha‐01). Electrical conductivity (EC) was measured using a digital conductivity meter. Available potassium (AK) was determined using a flame photometer (Pawar and Shah 2009), and available phosphorus (AP) was measured using Bray's method (P‐Bray) (Bray and Kurtz 1945). The Walkley and Black method was used to estimate soil organic carbon (SOC), while the micro‐Kjeldahl method was used to determine total nitrogen (TN) (Gupta 2000). The Soil chemical analyses were performed at Soil Water and Air Testing (SWAT) Laboratories Pvt. Ltd., Kathmandu, Nepal.

### 2.3 Statistical Analysis

To estimate species abundance, Daubenmire cover classes were converted to their respective midpoint values (e.g., 2.5% for class 1, 15% for class 2, 37.5% for class 3, 62.5% for class 4, 85% for class 5, and 97.5% for class 6) (Daubenmire 1959). Plant species were categorized into four abundance classes based on their cover: rare (< 25%), occasional (25%–50%), common (51%–75%), and abundant (> 75%) for further analysis (Oba et al. 2001). The midpoint cover values were also used as quantitative measures of species abundance for calculating diversity indices. Species richness was defined as the total number of species recorded per plot (Magurran 2004). To evaluate species diversity, both the Shannon and Simpson indices were calculated following Magurran (2004). The Shannon index (H′) was computed using the formula: −∑p*ᵢ* ln(p*ᵢ*), and the Simpson diversity index (1–D) was calculated as: 1 − ∑p*ᵢ* ^2^, where pᵢ represents the proportional cover of the *i*th species in a plot, calculated as p*ᵢ* = *nᵢ*/*N*, with *nᵢ* being the cover of a given species and *N* the total plant cover in the plot.

Prior to statistical analyses, all data were tested for normality and homogeneity of variance. Species richness data met the assumptions of normality, while biomass data did not; therefore, log transformation was applied where necessary (Feng et al. 2014).

To examine the effects of elevation and grazing intensity on species richness, plant height, and biomass, a linear mixed‐effects model was performed with site treated as a random factor using the *lme4* package in R 4.3.3 (R Core Team 2024). To assess the effects of grazing at each rangeland on species richness, biomass, and soil physicochemical properties—and to evaluate variation in plant life forms across grazing gradients—one‐way analysis of variance (ANOVA) was conducted. For biomass data in Khiriya rangeland and life‐form variability (specifically grasses) across all rangelands, the non‐parametric Kruskal–Wallis test was used due to the non‐normality of the data, even after transformation.

There is a well‐established premise that ecosystems with greater diversity tend to be more productive. To examine the relationship between species richness and biomass, a generalized linear model (GLM) was fitted, with species richness as the predictor variable and biomass as the response (McCullagh 2019). This analysis aimed to determine whether this relationship holds true within our study context. Since species richness represents count data, a Poisson error distribution with a logarithmic link function was used. To analyze changes in species composition along grazing gradients across the three rangelands, a similarity matrix was generated using the Bray‐Curtis index based on presence‐absence data. Therefore, no data transformation or standardization was applied before analysis. Non‐metric multi‐dimensional scaling (NMDS) was used to visualize compositional dissimilarities through an ordination biplot, implemented using the metaMDS function in the vegan package in R. A stress value < 0.2 was considered best fit for the accurate ordination, while a stress value (≤ 30) is also acceptable (Dexter et al. 2018). For pairwise comparisons within each group, we applied the *pairwise.adonis*() function. Prior to conducting PERMANOVA, the assumption of homogeneity of multivariate dispersion was tested using the *betadisper()* function in the vegan package, and the assumption was met. All analyses were performed in R (version 4.3.3; R Core Team 2024).

## 3 Results

### 3.1 Plant Lifeform, Species Richness and Diversity

Across the three rangelands, a total of 110 species of flowering plants belonging to 35 families and three pteridophytes belonging to three families were recorded (Table S1). Forbs were the most dominant life form (87.6%) followed by graminoids (8.8%), pteridophytes (2.6%), and shrubs (0.9%) across all sites and grazing intensities. Asteraceae was the largest family, accounting for 20% of total flowering plants, while leguminous plants accounted for only 2%. In all rangelands, forbs were significantly higher under moderately grazed (MG) and lightly grazed (LG) plots compared to the heavily grazed (HG) (Table 2). Particularly, in Dargari and Khali rangelands, forb richness increased along the grazing gradient, with the highest value recorded in non‐grazed (NG) plots. In contrast, forb richness in Khiriya rangeland did not show significant differences across grazing intensities. Meanwhile, graminoid species richness varied among rangelands, with MG and LG plots having higher graminoid richness. Shrubs were rare and observed only in Khiriya rangeland under NG plots, while pteridophytes were scarce and primarily limited to NG plots.

**TABLE 2:** Number of species (mean ± SD) by lifeform per plot (m^2^) in three rangelands across different grazing intensities.

Lifeform	Highly grazed	Moderately grazed	Lightly grazed	Non‐grazed
Dargari rangeland				
Forbs	5.67 ± 1.56A	10.75 ± 2.38B	11.33 ± 1.78B	9.25 ± 2.09B
Graminoids	1.33 ± 0.98A	2.58 ± 0.67B	1.75 ± 0.75A	1.67 ± 0.49A
Shrub	—	—	—	—
Pteridophytes	—	—	—	0.58 ± 0.50
Khali rangeland				
Forbs	3.61 ± 1.60A	9.51 ± 3.20B	7.12 ± 1.51B	16.80 ± 2.90C
Graminoids	1.51 ± 0.81A	2.42 ± 0.79B	2.81 ± 0.51B	3.22 ± 0.90B
Shrub	—	—	—	—
Pteridophytes	—	—	—	0.08 ± 0.3
Khiriya rangeland				
Forbs	8.25 ± 2.41A	10.25 ± 1.81A	10.61 ± 2.61A	9.01 ± 2.71A
Graminoids	1.00A	1.75 ± 1.14B	1.72 ± 0.81B	0.51 ± 0.51C
Shrub	—	—	—	0.08 ± 0.32
Pteridophytes	—	—	—	0.08 ± 0.31

*Note:* Different letters represent a significant difference among means across different grazing intensities at a 95% confidence interval (One‐way ANOVA).

**FIGURE 3:**
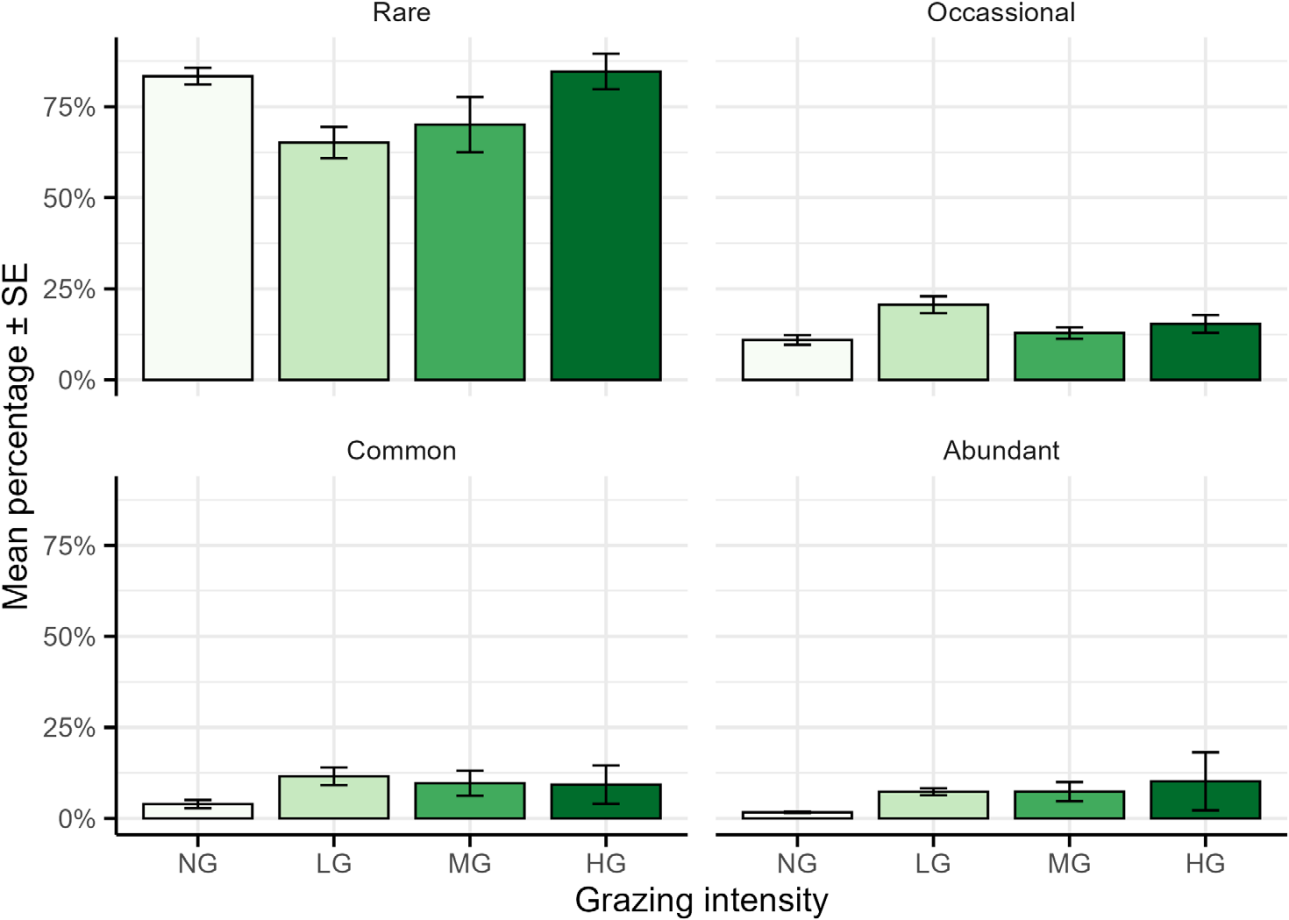
Percentage of species occurrence across four grazing intensities (HG, highly grazed; LG, lightly grazed; MG, moderately grazed; NG, non‐grazed). The error bars represent standard deviation.

Based on species cover, rare species (< 25% cover) were the most frequently recorded across grazing gradients, while abundant species (> 75% cover) were more common in the HG plots and least frequent in the NG plots (Figure 3).

**FIGURE 4:**
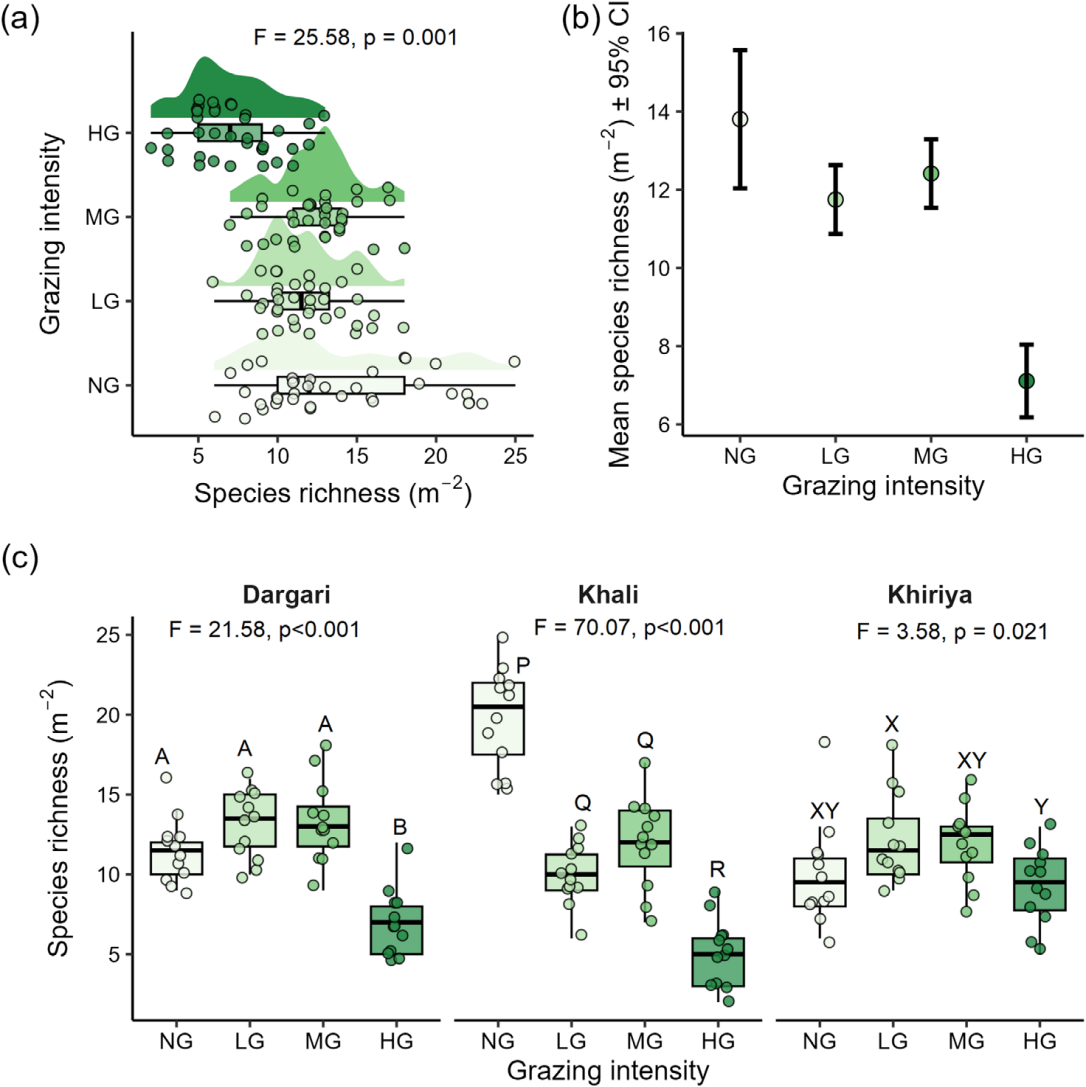
(a) Species richness across different grazing intensities (HG, highly grazed; LG, lightly grazed; MG, moderately grazed; NG, non‐grazed) of entire site, (b) Mean species richness ±95% CI, (c) Species richness across four grazing intensities in three rangelands. *p*‐values from one‐way ANOVA indicate significance at the 95% confidence level across different grazing intensity in each rangeland. Different upper‐case letters indicate significant differences (*p* < 0.05) based on post hoc Tukey HSD multiple comparisons among the four levels of grazing intensity within each site.

Species richness differed significantly across grazing gradients (*F* = 25.98, *p* < 0.001), with richness increasing from HG to NG plots. The NG plots had the highest number of average plant species (13.8 ± 5.3) while the HG had the lowest (7.1 ± 2.1). Moderately (12.4 ± 0.8) and lightly grazed (11.8 ± 1.7) plots had comparable species richness (Figure 4a,b). Elevation had no effect on species richness, except in NG plots, where species richness decreased with elevation (Estimate = −0.03, *p* < 0.001). Site (rangeland) as a random effect explained only a small portion (0.058%) of the remaining variance. Site‐specific patterns were seen in Dargaria and Khiriya rangelands, where species richness was high at MG followed by LG plots. In contrast, species richness was the highest in NG plots followed by MG in Khali. In Dargari, HG plots had lower species richness while LG or MG plots had higher species richness (Figure 4c). Nevertheless, in all rangelands, HG consistently supported the lowest species richness.

Overall, the Shannon diversity index was the highest under NG plots followed by MG, whereas the Simpson diversity index showed a more consistent pattern (> 0.9) across different grazing intensities (Table 3). Khali and Khiriya rangelands showed higher Shannon diversity values (H′ = 3.29) in NG plots compared to Dargari rangeland (H′ = 2.89). In Khiriya rangeland, both Shannon and Simpson indices were generally higher across all grazing intensities, with the highest Simpson index (0.96) recorded in NG plots. In Dargari rangeland, the Shannon index varied more across grazing intensities than the Simpson index (Table 3).

**TABLE 3:** Comparison of species diversity in three rangelands and the entire site across four grazing gradients.

Grazing intensity	Dargari rangeland		Khali rangeland		Khiriya rangeland		All rangeland	
	Shannon diversity index (H′)	Simpson diversity index (1–D)	Shannon diversity index (H′)	Simpson diversity index (1–D)	Shannon diversity index (H′)	Simpson diversity index (1–D)	Shannon diversity index (H′)	Simpson diversity index (1–D)
Highly grazed	2.73	0.90	2.25	0.87	2.88	0.95	2.62	0.90
Moderately grazed	2.89	0.93	3.11	0.94	3.07	0.95	3.02	0.94
Lightly grazed	3.02	0.94	2.56	0.89	2.95	0.93	2.8	0.92
Non‐grazed	2.89	0.93	3.29	0.94	3.29	0.96	3.16	0.94

### 3.2 Species Composition

**FIGURE 5:**
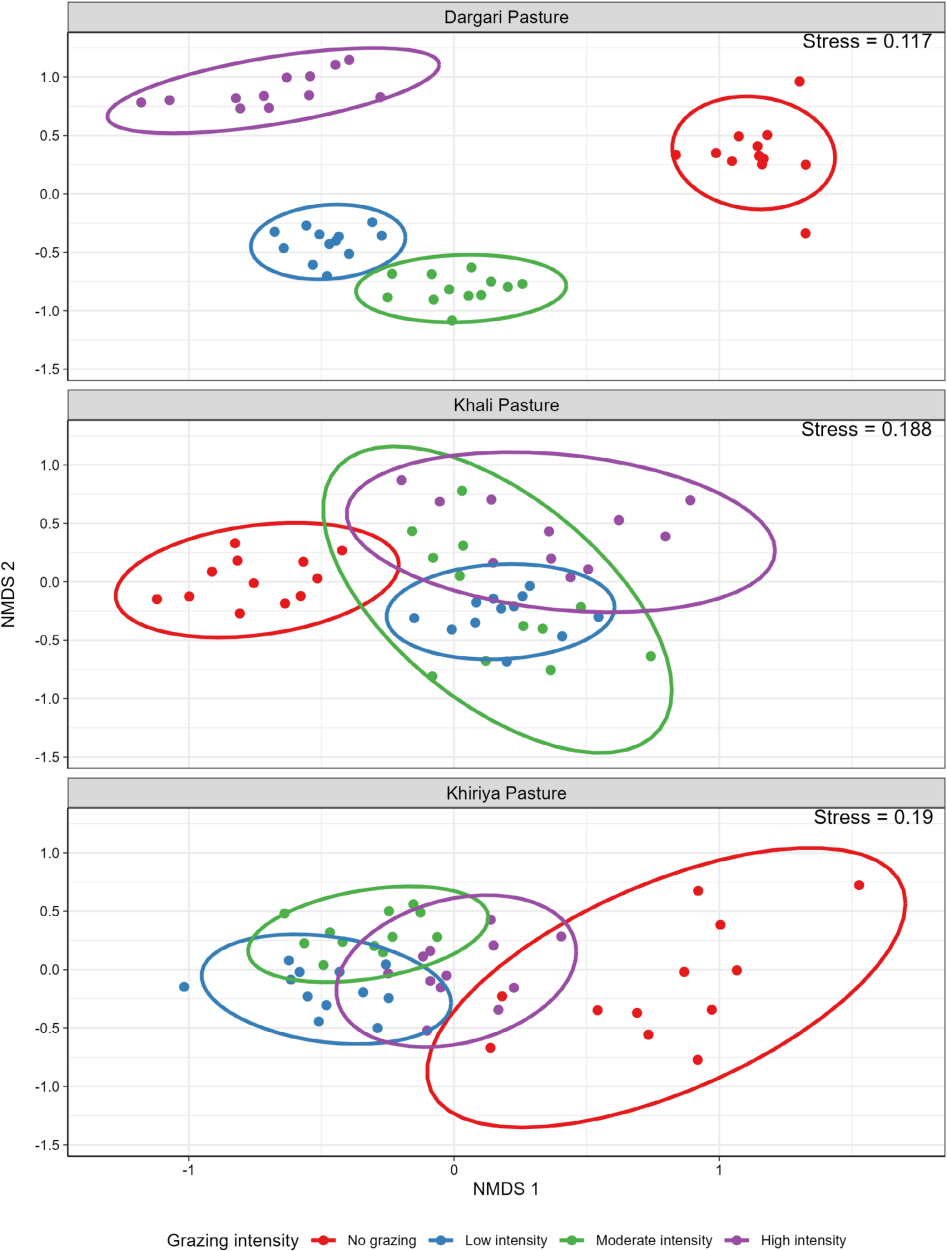
Non‐metric Multidimensional Scaling (NMDS) plot based on Bray–Curtis similarity matrix showing variation in plant species composition across grazing intensities. Each point represents a sampling plot; proximity indicates similarity in community composition.

Non‐metric multidimensional scaling (NMDS) analysis revealed that grazing intensity along three sites significantly influenced plant species composition, as also demonstrated in the ordination plot (Figure 5). The stress value for the NMDS was below 0.20 (i.e., 0.19), indicating a meaningful difference in community structure among grazing treatments across the three rangelands. The PERMANOVA analysis also revealed a significant impact of grazing intensity (*R* ^2^ = 0.15, *p* < 0.001) and sites (*R* ^2^ = 0.29, *p* < 0.001) as well as their interactions (*R* ^2^ = 0.69, *p* < 0.001). Similarly, pairwise comparisons also revealed significant differences (*p* < 0.01) in vegetation composition among all grazing intensity levels across the three study sites. However, the strength of compositional dissimilarity varied among sites. Dargari rangelands hold distinct community compositions (*R* ^2^ = 0.52–0.66) in comparison to the other two rangelands (Khali rangelands; *R* ^2^ = 0.23–0.51; Khiriya rangelands: *R* ^2^ = 0.20–0.40) (see Table S2). Overall, NG plots formed a distinct cluster. In contrast, MG and LG plots showed greater overlaps, suggesting transitional or shared species pools. But HG plots were more dispersed.

### 3.3 Plant Height

**FIGURE 6:**
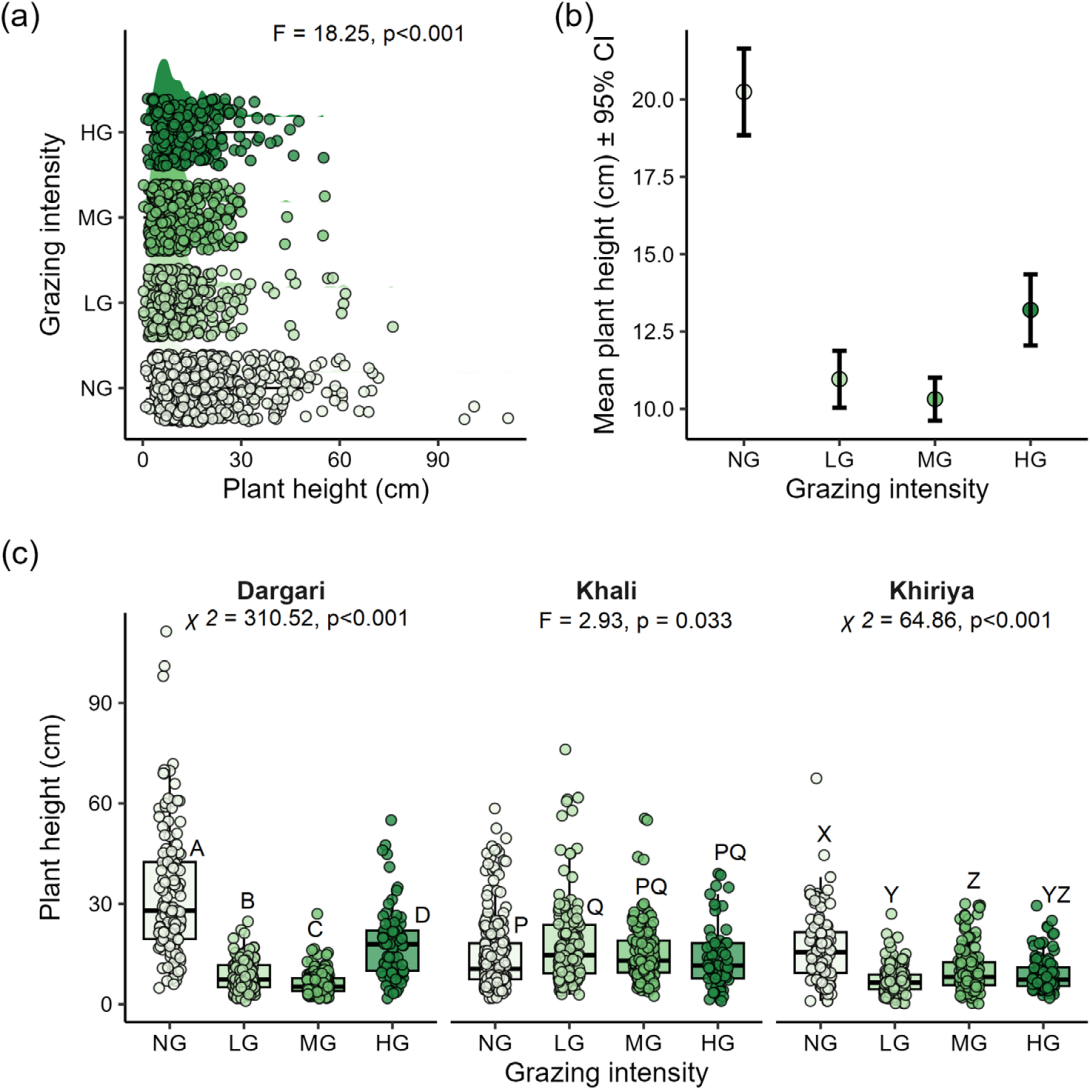
(a) Plant height across different grazing intensities (HG, highly grazed; LG, lightly grazed; MG, moderately grazed; NG, non‐grazed) of entire site, (b) Mean plant height ±95% CI, (c) Plant height across four grazing intensities (HG, highly grazed; LG, lightly grazed; MG, moderately grazed; NG, non‐grazed) in three rangelands. *p*‐values from one‐way ANOVA indicate significance at the 95% confidence level across different grazing intensity in each rangeland. Different upper‐case letters indicate significant differences (*p* < 0.05) based on post hoc Tukey HSD multiple comparisons among the four levels of grazing intensity within each site.

Linear mixed‐effects models showed that grazing intensity (*F* = 18.25, *p* < 0.001) and elevation (*F* = 119.08, *p* < 0.001) significantly affected plant structural traits (plant height). Plants in NG plots exhibited significantly greater height compared to those in HG plots (Estimate = 4.99 cm, *p* < 0.001). However, plant height in LG and MG plots did not differ from HG plots (*p* > 0.05). Site‐level variation accounted for a substantial proportion (41.7%) of the random effects, suggesting strong site‐level heterogeneity in plant height (Figure 6a–c).

### 3.4 Biomass Production

**FIGURE 7:**
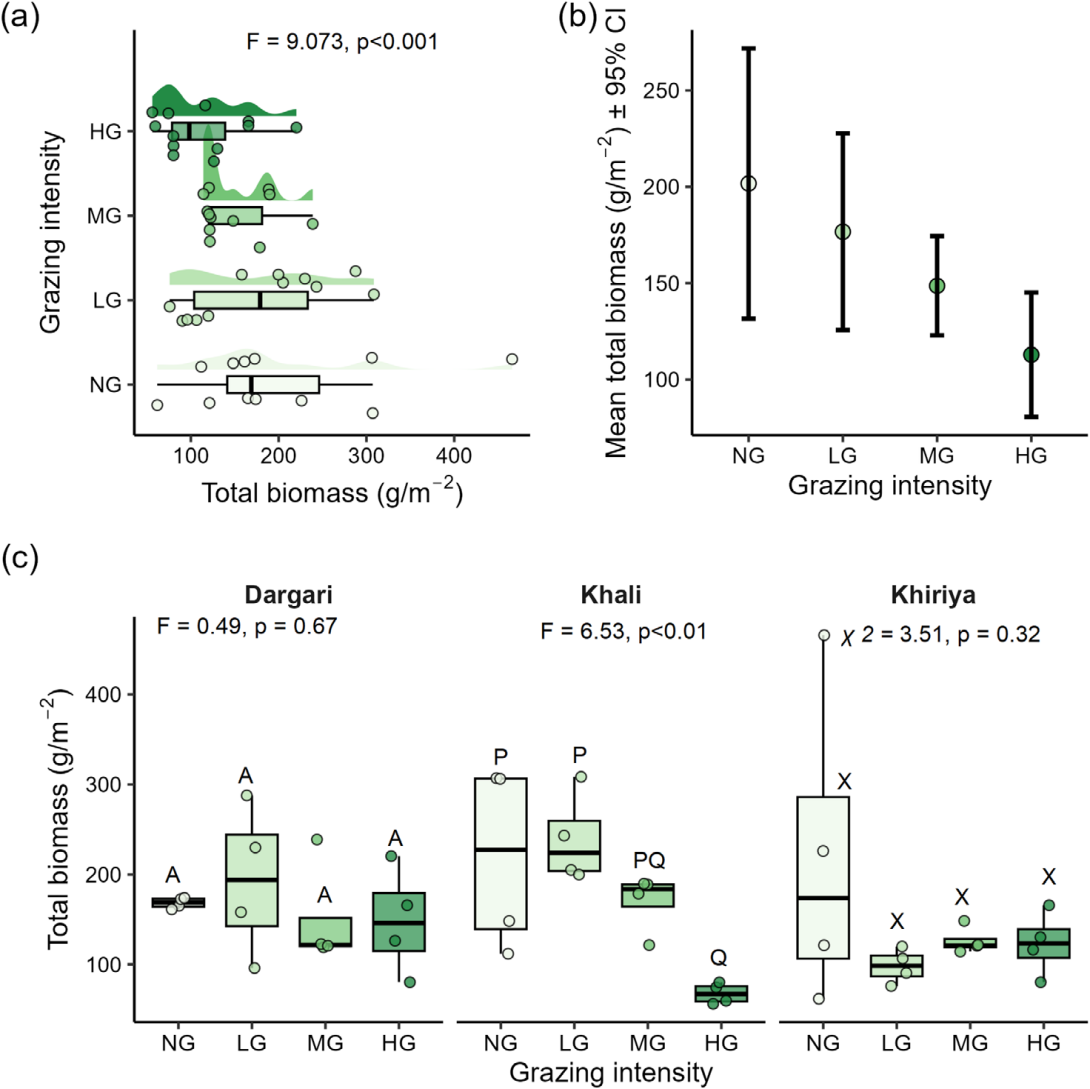
(a) Aboveground biomass across different grazing intensities (HG, highly grazed; LG, lightly grazed; MG, moderately grazed; NG, non‐grazed) of entire site, (b) Mean total biomass ±95% CI (c) Total aboveground biomass across four grazing intensities in three rangelands. *p*‐values from one‐way ANOVA indicate significance at the 95% confidence level across different grazing intensity in each rangeland. Different upper‐case letters indicate significant differences (*p* < 0.05) based on post hoc Tukey HSD multiple comparisons among the four levels of grazing intensity within each site.

Grazing intensity has a significant effect on aboveground biomass (*p* < 0.05), whereas elevation alone did not (*p* = 0.39). Patterns of biomass distribution were similar across all grazing intensities in Dargari and Khiriya (Figure 7c). However, the interaction effect between elevation and grazing intensity on total biomass was significant (*p* < 0.05). Specifically, total biomass was the highest in NG plots (*t* = 2.639, *p* = 0.0093), followed by LG plots (*t* = 2.315, *p* = 0.0221), while HG plots had the lowest biomass (*t* = −0.602, *p* = 0.54) (Figure 7a–c). Site‐level variability accounted for only 8.4% of the total variance (SD = 21.68), with the majority (91%) explained by within‐site differences.

### 3.5 Plant Species Richness and Biomass Relationship

**FIGURE 8:**
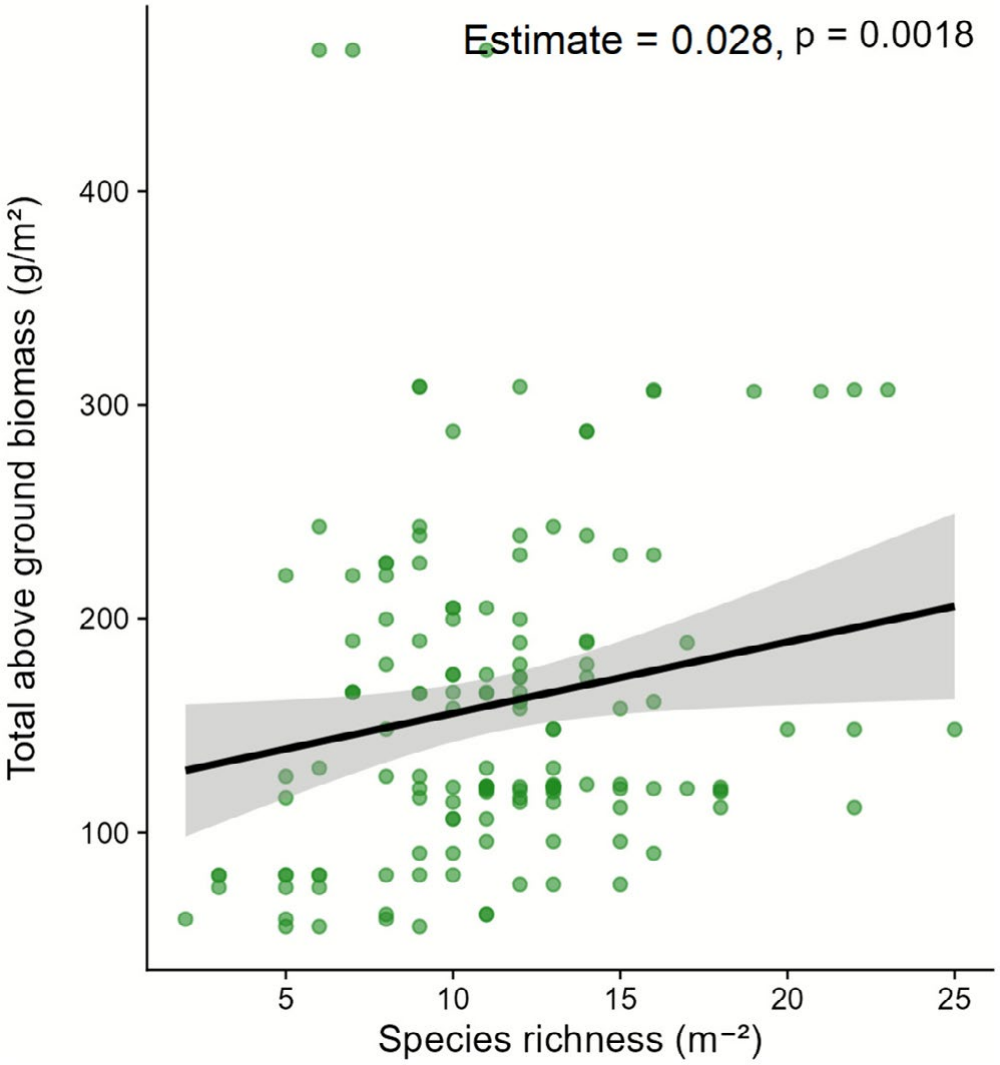
The relationship between plant species richness and aboveground plant biomass across all the sampling sites. The fitted lines represent generalized linear models (GLMs) illustrating the trend.

GLM analysis revealed a significant positive relationship between overall plant species richness and total above‐ground biomass (*p* = 0.0018) (Figure 8). The model predicted that with a unit increase in species richness, the biomass production was expected to increase by about 2.9%.

### 3.6 Soil Physicochemical Properties

**FIGURE 9:**
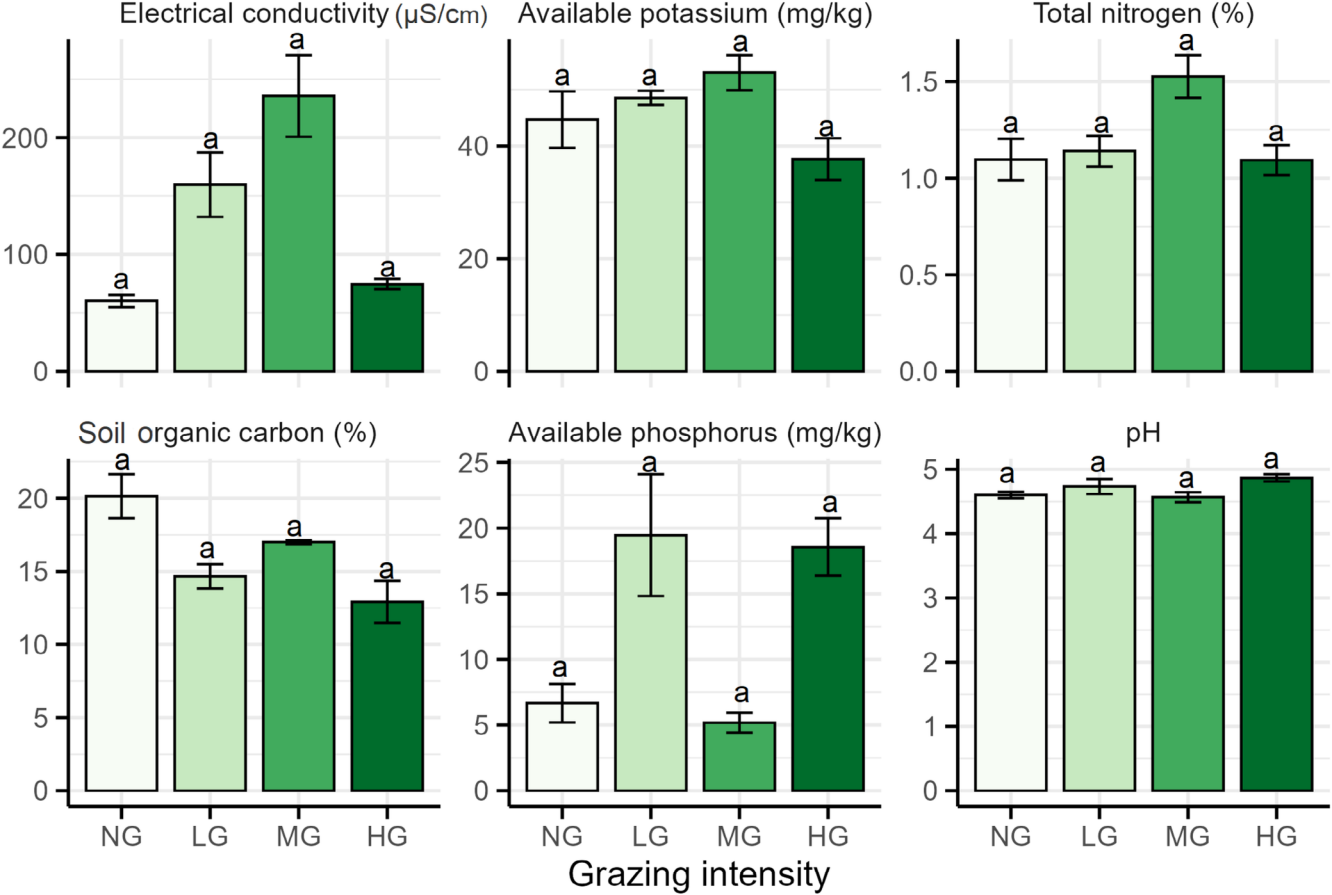
Soil chemical properties across four grazing gradients (HG, highly grazed; LG, lightly grazed; MG, moderately grazed; NG, non‐grazed). Different lower‐case letters indicate significant differences (*p* < 0.05) based on post hoc Tukey HSD multiple comparisons among the four levels of grazing intensity.

Soil pH did not vary across grazing gradients at any of the three sites (*p* > 0.05). Soil remained consistently acidic with pH values ranging from 4 to 5.7 across the grazing gradients. Electrical conductivity (EC) was significantly higher in MG plots at Khali rangeland, whereas soil organic carbon (SOC) was the highest in NG plots at Dargari and Khali rangelands but peaked in MG plots at Khiriya rangeland. Total nitrogen (TN) was also higher in MG plots at Khiriya and Khali rangelands but lower at Dargari rangeland under the same grazing intensity. Available phosphorus (AP) content was notably low in Khiriya rangeland, without noticeable variation across grazing gradients, whereas LG plots in Khali rangeland had the highest phosphorus levels. Available potassium (AK) levels were higher in LG and NG plots at Khiriya and Dargari rangelands, whereas MG plots in Khali rangeland had the highest K levels (Figure 9).

In addition, correlation analysis revealed positive correlations between AGB and some soil properties [EC (*r* = 0.38, *p* < 0.01), SOC (*r* = 0.201, *p* < 0.05) and AK (*r* = 0.19, *p* < 0.05)]. Furthermore, SR was found to be positively correlated with SOC (*r* = 0.23, *p* < 0.01) and AP (*r* = 0.51, *p* < 0.001) while negatively correlated with soil pH (*r* = −0.45, *p* < 0.001) (Table 4).

**TABLE 4:** Correlation matrix of eight soil parameters.

	ABG	SR	pH	EC	SOC	AK	TN	AP
AGB	1							
SR	0.19*	1						
pH	0.0084	−0.45***	1					
EC	0.38***	0.059	0.26**	1				
SOC	0.201*	0.23**	0.23**	0.15	1			
AK	0.19*	−0.068	−0.017	0.51***	0.069	1		
TN	0.065	−0.093	0.59***	0.46***	0.31***	0.28***	1	
AP	0.085	−0.51***	0.701***	0.45***	0.34***	0.35***	0.35***	1

*Note:* Correlation is significant * at the 0.05 level; ** at the 0.01 level; *** at the 0.001 level.

Abbreviations: AGB, aboveground biomass; AK, available potassium; AP, available phosphorus; EC, electrical conductivity; pH, soil pH; SOC, soil organic carbon; SR, species richness; TN, total nitrogen.

## 4 Discussion

Understanding the ecological impacts of livestock grazing on rangeland conditions is essential for sustainable rangeland management. While numerous studies have shown that both heavy grazing (overgrazing) or complete grazing exclusion (no grazing) can lead to rangeland degradation and moderate grazing is often associated with positive biodiversity outcomes (Pokharel et al. 2007; Wang et al. 2026), these effects are highly context‐dependent. In the unique ecological context of sub‐alpine rangelands of Nepal, determining site‐specific impacts of grazing is more critical for sustainable rangeland management. This study evaluated the influence of varying grazing intensities on plant lifeform, species diversity, community structure, biomass production, and plant height in sub‐alpine rangelands of western Nepal.

### 4.1 Impacts on Plant Lifeform, Species Richness and Diversity

Grazing intensity had a significant effect on the distribution of plant lifeforms, species richness, and diversity. Across all rangeland sites, forbs were more abundant than graminoids, indicating a shift in community composition and potential implications for forage quality. Although many forbs are palatable to sheep, selective removal of preferred graminoids, together with competitive release and trait‐based tolerance (e.g., chemical/structural defenses, deep root systems), can increase the relative abundance of grazing‐tolerant forb species under sustained grazing and trampling despite their higher compensatory growth capacity (Jones 2000; Hawkes and Sullivan 2001). Thus, the observed forb dominance reflects compositionally specific responses to grazing rather than a uniform lack of palatability across forbs. This also aligns with our site data (lower graminoid cover in several sites) and with studies showing that prolonged grazing disturbances such as trampling, uprooting, and digging lead to the competitive exclusion of dominant graminoids while creating a niche for the establishment or persistence of diverse forb assemblages (Bråthen et al. 2021).

Species richness increased along the grazing gradient from HG to NG, with MG plots often supporting the highest number of species, indicating that moderate grazing may reduce competitive exclusion, facilitating coexistence among species. This aligns with the findings that moderate grazing reduces competitive exclusion, preventing dominance of a few competitive species, allowing more species to coexist (Holechek et al. 2006; Zhang, Wuriliga, et al. 2023). The greater proportion of dominant species (species with > 75% cover) in HG plots indicates plant community simplification, a common sign of degradation under intense grazing pressure (Kassahun et al. 2009). Under intensive grazing, few non‐palatable or less palatable species like *Pteridium revolutum*, *Koenigia mollis*, *Rumex nepalensis*, *Salvia campanulata* are likely to increase their abundance at the cost of palatable species, especially grasses. In contrast, MG appeared to suppress dominance and promote evenness by enabling coexistence of both rare and common species. Furthermore, Shannon diversity was the highest under NG and MG plots, whereas Simpson's index showed less variation. Previous studies also report that herbivory tends to reduce diversity through selective feeding of more preferred species (Tegegn et al. 2010; Wan et al. 2015).

Interestingly, across all three rangeland sites, comparable levels of diversity were observed among the different grazing intensity plots. This pattern may reflect the legacy of past grazing, where historical factors such as seed bank composition and residual soil nutrient status continue to shape the present community structure. Although the precise grazing history of the non‐grazed (NG) plots is unknown, these areas may currently be in a resting phase, which could have contributed to the observed diversity patterns. Similarly, the comparable diversity levels in NG, MG, and LG plots may be attributed to the relatively short duration of grazing exclusion, during which vegetation may not yet have fully transitioned between states, as well as to shared environmental conditions that may mask grazing effects. In this context, early grazing exclusion may also be linked to a decline in herd size or changes in management practices, consistent with the reported reduction in livestock numbers in Patarasi Rural Municipality (National Statistical Office (NSO) 2021). Meanwhile, grazing effects were less evident overall, except for the more pronounced impacts observed in heavily grazed (HG) plots located near herders' shelters. Comparable results were also reported by Wang et al. (2017), who found similar diversity patterns across grazing intensities. This suggests the strong influence of local environmental conditions in shaping rangeland species richness and diversity, which may override the effects of grazing intensity and challenge the universal applicability of the Intermediate Disturbance Hypothesis (IDH). Site‐specific differences were also evident among the studied rangelands; for instance, average diversity in Dargari was lower than in the other two sites. Dargari, situated at higher elevation and under drier conditions, likely supports lower plant diversity. These findings highlight that local environmental context and grazing history jointly mediate plant community responses to grazing intensity.

Overall, high‐intensity grazing negatively affects species diversity, while moderate to low‐intensity grazing maintains higher diversity through the balance mechanism between disturbance and resource availability. These findings support the intermediate disturbance hypothesis (Connell 1978), which posits that species diversity peaks at intermediate levels of disturbance due to a balance between colonization and competitive exclusion, as shown in other studies (Bhattarai et al. 2004; Thapa et al. 2016). Moderate grazing may allow resting time for vegetation regeneration and root system regeneration between grazing cycles (Jones 2000), making it optimal for sustaining rangeland biodiversity. Thus, these findings highlight the importance of adaptive grazing management strategies such as rotational grazing and periodic resting that allow vegetation to recover while balancing forage production with biodiversity conservation (Aryal et al. 2015).

### 4.2 Differences in Species Composition

The NMDS results showed clear variation in species composition across grazing intensities in three rangelands. The three rangeland sites differ in magnitude of response, possibly due to local environmental heterogeneity (microclimate, soil, grazing animal types). In addition, unique species composition along different grazing gradients may indicate a significant impact of grazing intensity and spatial variation on species composition. NG plots formed distinct clusters, likely due to the presence of grazing‐sensitive species. In contrast, MG and LG plots showed overlapping clusters, indicating possible coexistence of tolerant and sensitive species. HG plots were more dispersed, reflecting altered community structure under high grazing pressure. Large differences occurred between NG and HG plots in all sites, indicating that both grazing exclusion and high grazing pressure create distinct species assemblages. These patterns align with previous studies reporting compositional shifts with grazing intensity. Selective grazing often reduces palatable perennial grasses, allowing competitive release of less‐palatable, weedy, or thorny species (Oba et al. 2001; Mugabe et al. 2017). For instance, non‐grazed plots in Upper Mustang, Nepal, were found dominated by highly palatable species (Pokharel 2005), while in Ethiopia, heavy grazing favored annual grass, drought‐tolerant species, and forbs, whereas reduced grazing supported desirable perennial grasses (Sisay and Baars 2002; Gebremeskel 2005; Kassahun et al. 2009; Tegegn et al. 2010). Thus, these results highlight the consistent yet site‐specific impact of grazing intensity on rangeland vegetation structure.

Species with similar ecological niches often co‐occur, but their abundance shifts with grazing intensity depending on functional traits and resource competition (Huang et al. 2019). Plants with similar functional traits may share comparable microhabitats or be under similar grazing pressures, leading to their co‐occurrence (Gaujour et al. 2012). In addition to selective species removal, changes in microhabitat conditions caused by trampling and nutrient deposition, particularly near water points, may also facilitate fast‐growing annuals (Tegegn et al. 2010). Beyond altering species composition, grazing also affects forage quality, litter decomposition, and other ecosystem processes (Wu et al. 2008; Tegegn et al. 2010). Therefore, managing grazing intensity is not only crucial for preserving plant diversity but also for sustaining rangeland productivity and ecological function.

### 4.3 Impacts of Grazing on Plant Height

Plant height is a key functional trait reflecting growth performance and competitive ability, particularly for light acquisition (Díaz et al. 2007). We found significantly taller plants in non‐grazed plots than in heavily grazed ones, consistent with studies showing that grazing suppresses vertical growth and reduces competitiveness for light and resources (Grime 2006; Rahmanian et al. 2019; Jäschke et al. 2020; Wang et al. 2021, 2023; Avramidou et al. 2024). Interestingly, plant height in MG and LG plots did not differ significantly from HG plots, suggesting that even moderate grazing can suppress taller species due to defoliation and trampling (Ostoja et al. 2014; Avramidou et al. 2024). Additionally, elevation also had a negative effect on plant height, likely due to harsher climatic conditions and shorter growing seasons at higher altitudes, where shorter stature offers protection from extreme weather (Rasmann et al. 2014; Pandey et al. 2021). Significant variation in plant height across the three rangelands further emphasizes the role of site‐specific factors such as microclimate, soil fertility, and grazing history (Bloor and Pottier 2014). These findings suggest a complex interplay between grazing intensity, elevation, and local ecological conditions in shaping plant structure and growth.

### 4.4 Impacts of Grazing on Biomass Production

Biomass production was the highest in NG plots, followed by LG plots and the lowest in HG plots. This pattern indicates that grazing pressure reduces aboveground biomass through defoliation and trampling. The result is consistent with earlier studies reporting significant biomass declines under intense grazing (Oba et al. 2001). In Nepal's Trans‐Himalayan rangelands, aboveground biomass during the peak growing season was also found to be highest in ungrazed plots compared to grazed ones (Pokharel et al. 2007). However, grazing intensity alone does not fully explain rangeland productivity, which also depends on climatic, edaphic, and topographic factors as well as vegetation characteristics (Zaady et al. 2001; Yan et al. 2013; Xiong et al. 2014; Lu et al. 2017; Ahmed et al. 2020). For instance, inter‐annual precipitation variation strongly influences above‐ground biomass (Bat‐Oyun et al. 2016), while temperature during the growing season affects plant growth in arid and semi‐arid environments (Carmona et al. 2013; Ahmed et al. 2020). In this study, the negative effects of grazing were more pronounced at higher elevations, likely due to lower temperatures that shift plant resource allocation toward belowground growth more than aboveground production (Poorter et al. 2012).

Types of lifeforms showed limited association with biomass differences across grazing gradients, suggesting that functionally similar groups may respond uniformly to grazing pressure, due to shared niches or exposure (Škornik et al. 2010). Moreover, local conditions such as soil properties and microclimate significantly influenced biomass production (Xu et al. 2014; Lu et al. 2017). Our findings highlight a complex interplay between grazing intensity, elevation, and site‐specific vegetation characteristics in shaping rangeland productivity.

### 4.5 Relationship Between Species Richness and Biomass Production

A positive relationship was observed between biomass production and species richness, suggesting that richer plant diversity supports higher biomass production, a key driver of ecosystem functioning (Ingty 2021). Similar patterns have been reported globally, with species richness often increasing alongside biomass (Hector 1998; Sonkoly et al. 2019). This pattern may result from multiple factors. First, diverse plant communities utilize resources more efficiently due to greater niche differentiation and complementary traits (Sonkoly et al. 2019). Second, facilitating interactions such as nitrogen‐fixing legumes can improve soil fertility, supporting the establishment of other species in nutrient‐poor environments (Olsen et al. 2013; Kelemen et al. 2015). However, in our context, no such pattern was observed as overall legume richness was low. Perhaps the statement was intended to convey the general principle that higher species richness increases the likelihood of including highly productive species (Kelemen et al. 2015). However, high productivity is not always linked to high species richness. For instance, areas dominated by perennial species may produce high biomass despite low diversity, and high functional evenness can sometimes reduce biomass production (Sonkoly et al. 2019). Furthermore, the biomass‐species relationship can vary with scale; it may follow a unimodal (hump‐shaped) pattern, with both peaking under intermediate conditions and even showing an inverse pattern, depending on spatial scales (Guo and Berry 1998; Mittelbach et al. 2001). Thus, the positive local‐scale relationship observed here may represent one part of a broader, hump‐shaped regional trend. At high productivity levels, once a certain threshold is exceeded, competitive exclusion by dominant species can lead to a decline in species diversity (Mittelbach et al. 2001; Bhandari and Zhang 2019).

### 4.6 Impact of Grazing on Soil Physicochemical Properties

Soil physiochemical properties are key determinants of rangeland productivity and are heavily influenced by grazing intensity (Li et al. 2018). Soils across all studied rangelands were acidic, potentially limiting legume abundance, also reflected in the low occurrence of leguminous species across all grazing gradients (Marschner 2011). Soil organic carbon (SOC) was highest in NG plots at Dargari and Khali, and in MG plots at Khiriya, suggesting that lower grazing pressure supports higher litter input and carbon accumulation. This pattern can be further explained by the distribution of dung deposition and the degree of trampling, both of which are closely associated with grazing intensity (Tate et al. 2003). In grazed plots, nutrients are returned to the soil through dung and urine deposition, whereas in ungrazed plots, the standing vegetation contributes itself to soil nutrients through litter decomposition (Haynes and Williams 1993). However, in heavily grazed plots, excessive trampling leads to vegetation loss and soil compaction, thereby reducing water infiltration and increasing nutrient leaching, ultimately leading to lower soil nutrient availability, specifically SOC (Li et al. 2021). Similar trends have been reported in alpine meadows of the Qinghai‐Tibetan Plateau, where moderate grazing enhanced SOC, while heavy grazing reduced both SOC and TN (Li et al. 2018). Some other studies also suggest that low levels of grazing intensity can enhance SOC and TN due to stimulated microbial activity and nutrient cycling (Gao et al. 2007; Wei et al. 2011). In this study, TN was elevated in MG plots at Khiriya and Khali, potentially due to balanced disturbance and nutrient cycling. However, low TN under similar grazing intensity at other sites reflects the influence of site‐specific factors, as reported in Chinese semi‐arid rangelands (Xu et al. 2014). Potassium availability (AK) varied by site and grazing intensity, with higher levels in LG and NG plots at Khiriya and Dargari, and in MG at Khali, similar patterns observed in the meadow steppes of China by Wang et al. (2022). Correlation analysis revealed significant associations between above‐ground biomass production, species richness, and key soil properties, indicating the important role of soil conditions in supporting species diversity and plant productivity in sub‐alpine rangelands. Previous studies have also shown that high nutrient availability increases species diversity and biomass production by improving soil structure, nutrient cycling, and water retention (Guo et al. 2022; Xu et al. 2022). SOC is a key factor in soil fertility as it enhances nutrient availability and microbial activity, supporting extensive plant growth (Magdoff and Van Es 2021).

Generally, high acidity (low pH ~4.4) is associated with lower diversity and productivity (Somavilla et al. 2021). Interestingly, this study found a negative association between species richness and soil pH, indicating that slightly acidic soils may favor greater species richness. One possible explanation is that low pH reduces competition by limiting the dominance of species adapted to alkaline conditions. Other studies have shown that deviation from an optimal pH, either increasing or decreasing, can reduce richness (Palpurina et al. 2017). Overall, these results suggest that soil physicochemical properties play an important role in regulating ecosystem dynamics and productivity of rangeland ecosystems.

Nevertheless, since the study was conducted during a single growing season, the observed grazing–diversity–biomass relationships should be interpreted with caution. Long‐term monitoring would be valuable for capturing interannual variability and improving our understanding of temporal dynamics. In addition to grazing intensity as a form of disturbance, factors such as grazing duration, timing (season), livestock type, and local environmental conditions are also critical in determining the ecological health of rangelands. Considering these factors in future studies would therefore provide more meaningful insights.

## 5 Conclusion

This study demonstrates that grazing intensity strongly influences plant community structure, diversity, biomass productivity, and soil chemical properties in the sub‐alpine rangelands of Jumla district, western Nepal. High grazing intensity consistently reduced species richness, biomass, and plant height, while promoting dominance by a few tolerant species. In contrast, moderate grazing intensity was found to support greater species diversity, higher biomass productivity, and a balanced coexistence of both grazing‐sensitive and tolerant species, aligning with the intermediate disturbance hypothesis. Soil properties, particularly soil organic carbon and total nitrogen, were also more favorable under moderate and light grazing, although site‐specific environmental conditions played a critical role in modulating these effects. The observed positive relationship between species richness and biomass productivity highlights the functional importance of maintaining plant diversity in rangeland ecosystems. However, elevation did not have a significant impact on species richness or biomass production, but it did have an impact on plant height. This indicates a strong environmental control on plant height under varying grazing pressures. Furthermore, the site‐specific influence of grazing intensity highlights the role of microclimatic factors in shaping rangeland diversity and productivity. Thus, our findings suggest that neither heavy grazing nor complete exclusion is optimal for healthy rangeland ecosystems. Instead, site‐specific, rotational, or seasonal resting schemes could serve as practical strategies to operationalize moderate grazing for sustainability. Such approaches, when implemented with thoughtful consideration of environmental controls, can help sustain biodiversity, maintain ecosystem functions, and support pastoral livelihoods.

## Author Contributions

**Tulasa Chaudhary:** conceptualization (supporting), formal analysis (lead), investigation (lead), methodology (supporting), visualization (lead), writing – original draft (lead). **Uttam Babu Shrestha:** conceptualization (lead), formal analysis (supporting), methodology (lead), project administration (lead), supervision (lead), writing – original draft (supporting), writing – review and editing (lead). **Uma Dhungel:** conceptualization (supporting), formal analysis (supporting), methodology (supporting), writing – review and editing (supporting). **Nishan Baral:** conceptualization (supporting), formal analysis (supporting), investigation (supporting), writing – review and editing (supporting). **Bharat Babu Shrestha:** writing – original draft (supporting), writing – review and editing (supporting). **Shikui Dong:** conceptualization (supporting), writing – review and editing (supporting).

## Funding

This work was supported by the National Science Foundation of China [Grant number: 32361143870] and the USAID Biodiversity (Jal‐Jangal) project in Nepal [Grant number: PUR‐KTM‐24‐0061].

## Conflicts of Interest

The authors declare no conflicts of interest.

## Supporting information


**Table S1:** Summary of all plant taxa captured during the study (γ‐diversity) and their coverage.

**Table S2:** Summary table showing (1) stress values from NMDS, (2) PERMANOVA results.

## Data Availability

The data that supports the findings of this study is available in the Supporting Information of this article.
