# Supplementary material for: Grazing Intensity Shapes Vegetation Structure and Soil Characteristics in High‐Elevation Rangelands of Nepal
Source: Ecol Evol. 2025 Dec 17;15(12):e72689. doi: 10.1002/ece3.72689 (PMC12711437; doi:10.1002/ece3.72689)
Supplement: Supplementary file 1 — Table S1: Summary of all plant taxa captured during the study (γ‐diversity) and their coverage. [file ECE3-15-e72689-s002.docx]

**Supplementary Table S1: Summary of all plant taxa captured during the study (γ-diversity) and their coverage**

| **Family** | **Scientific Name** | **Grazing intensity** | | | |
| --- | --- | --- | --- | --- | --- |
|  |  | **NG** | **LG** | **MG** | **HG** |
| **Dargari** | | | | | |
| Apiaceae | *Bupleurum longicaule* Wall. ex DC. | 0 | 83.33 | 0 | 0 |
|  | *Cortia depressa* (D.Don) C.Norman | 0 | 8.33 | 83.33 | 0 |
| Araceae | *Arisaema utile* Hoo.f. ex Engl. | 0 | 0 | 0 | 8.33 |
| Asparagaceae | *Polygonatum singalilense* H. Hara | 0 | 0 | 0 | 8.33 |
| Asteraceae | *Anaphalis busua* (Buch.-Ham. ex D. Don) DC. | 25 | 83.33 | 0 | 0 |
|  | *Anaphalis contorta* (D. Don) Hook.f. | 0 | 41.67 | 0 | 0 |
|  | *Anaphalis nepalensis* (Spreng.) Hand.-Mazz. | 0 | 0 | 58.33 | 0 |
|  | *Artemisia indica* Willd. | 0 | 0 | 0 | 8.33 |
|  | *Cremanthodium arnicoides* (Dc. ex Royle) R.D. Good | 75 | 16.67 | 0 | 8.33 |
|  | *Dubyaea hispida* DC. | 8.33 | 75 | 0 | 33.33 |
|  | *Hippolytia longifolia* (Rech.f.) C.Shih | 0 | 83.33 | 91.67 | 0 |
|  | *Jacobea raphanifolia* (Wall. ex Dc.) B. Nord. | 0 | 91.67 | 0 | 75 |
|  | *Jurinea* sp. | 0 | 0 | 16.67 | 0 |
|  | *Leontopodium himalayanum* DC. | 0 | 50 | 8.33 | 0 |
|  | *Melanoseris lessertiana* (DC.) Decne. | 0 | 66.67 | 16.67 | 0 |
|  | *Oreoseris nivea* DC. | 0 | 0 | 83.33 | 16.67 |
|  | *Saussurea candolleana* (DC.) Sch.Bip. | 41.67 | 0 | 0 | 0 |
|  | *Saussurea hieracioides* Hook.f. | 0 | 83.33 | 83.33 | 0 |
|  | *Senecio candolleanus* Wall. ex DC. | 33.33 | 0 | 0 | 0 |
| Balsaminaceae | *Impatiens stenantha* Hook.f. | 16.67 | 0 | 0 | 0 |
| Boraginaceae | *Hackelia uncinata* (Royle ex Benth.) C.E.C.Fisch. | 33.33 | 0 | 0 | 16.67 |
| Campanulaceae | *Cyananthus lobatus* Wall. ex Benth. | 0 | 66.67 | 33.33 | 0 |
| Caprifoliaceae | *Morina nepalensis* D.Don | 0 | 41.67 | 16.67 | 0 |
|  | *Nardostachys jatamansi (D.Don) DC.* | 83.33 | 0 | 0 | 0 |
| Crassulaceae | *Rhodiola wallichiana* (Hook.) S.H.Fu | 83.33 | 0 | 0 | 0 |
| Cyperaceae | *Carex nubigena* D.Don | 0 | 50 | 41.67 | 58.33 |
|  | *Carex* sp.1 | 0 | 16.67 | 66.67 | 8.33 |
|  | *Carex* sp.2 | 50 | 0 | 0 | 16.67 |
|  | *Carex unciniiformis* Boeckeler | 75 | 100 | 100 | 0 |
| Dryopteridacee | *Polystichum bakerianum* (Ark. ex C.B. Clarke) Diels | 50 | 0 | 0 | 0 |
| Euphorbiaceae | *Euphorbia stracheyi* Boiss. | 0 | 0 | 83.33 | 0 |
| Gentianaceae | *Halenia elliptica* D.Don | 0 | 8.33 | 0 | 0 |
|  | *Swertia cuneata* Wall. ex G. Don | 0 | 8.33 | 25 | 0 |
| Geraniaceae | *Geranium donianum*Sweet | 16.67 | 0 | 0 | 8.33 |
|  | *Geranium wallichianum* D.Don ex Sweet | 16.67 | 8.33 | 0 | 25 |
| Lamiaceae | *Elsholtzia pilosa* (Benth.) Benth. | 0 | 0 | 0 | 8.33 |
|  | Unidentified sp2 | 0 | 0 | 0 | 8.33 |
| Nartheciaceae | *Aletris* sp. | 0 | 0 | 8.33 | 0 |
| Onagraceae | *Epilobium brevifolium* D.Don | 33.33 | 16.67 | 16.67 | 0 |
| Orobanchaceae | *Pedicularis gracilis* Wall. ex Benth. | 0 | 41.67 | 58.33 | 0 |
|  | *Pedicularis hoffmeisteri* Klotzsch | 0 | 8.33 | 0 | 0 |
| Papaveraceae | *Corydalis juncea* Wall. | 50 | 0 | 0 | 0 |
|  | *Meconopsis* sp. | 8.33 | 0 | 0 | 0 |
| Plantaginaceae | *Veronica himalensis* D. Don | 0 | 16.67 | 0 | 0 |
| Poaceae | *Poa rajbhandarii* Noltie | 0 | 0 | 0 | 50 |
|  | *Tenaxia cumminsii* (Hook.f.) N.P.Barker & H.P.Linder | 33.33 | 8.33 | 50 | 0 |
| Polygonaceae | *Bistorta affinis*(D.Don) Greene | 8.33 | 8.33 | 66.67 | 0 |
|  | *Koenigia nepalensis D.Don* | 0 | 0 | 8.33 | 8.33 |
|  | *Persicaria nepalensis* (Meisn.) H. Gross | 0 | 0 | 0 | 83.33 |
|  | *Koenigia mollis* (D.Don) T.M.Schust. & Reveal | 91.67 | 0 | 0 | 0 |
|  | *Rumex nepalensis* Spreng. | 0 | 0 | 0 | 100 |
| Polypodiaceae | *Polystichum prescottianum* (Wall. ex Mett.) T. Moore | 16.67 | 0 | 0 | 0 |
| Primulaceae | *Primula denticulata* Sm. | 0 | 0 | 8.33 | 0 |
| Ranunculaceae | *Anemonastrum polyanthes* (D.Don) Holub | 8.3 | 0 | 0 | 8.33 |
|  | *Eriocapitella rivularis* (Buch.-Ham. ex DC.) Christenh. & Byng | 0 | 0 | 0 | 8.33 |
|  | *Ranunculus brotherusii* Freyn | 0 | 8.33 | 16.67 | 25 |
| Rosaceae | *Astragalus* sp. | 0 | 0 | 0 | 16.67 |
|  | *Cotoneaster microphyllus* Wall. ex Lindl. | 8.33 | 0 | 0 | 0 |
|  | *Fragaria nubicola* (Lindl. ex Hook.f.) Lacaita | 91.67 | 0 | 0 | 8.33 |
|  | *Geum elatum* Wall. f. rubrum Ludlow | 75 | 0 | 66.67 | 0 |
|  | *Potentilla griffithii* Hook.f. | 0 | 83.33 | 58.33 | 75 |
|  | *Potentilla lineata*  Trevir. | 0 | 0 | 16.67 | 0 |
|  | *Sibbaldia cuneata* Edgew. | 0 | 83.33 | 75 | 0 |
| Saxifragaceae | *Saxifraga parnassifolia* D.Don | 66.67 | 0 | 75 | 0 |
|  | *Saxifraga* sp1 | 50 | 0 | 0 | 0 |
| Violaceae | *Viola biflora* L. | 0 | 50 | 0 | 0 |
| **Khali** | | | | | |
| Apiaceae | *Bupleurum longicaule* Wall. ex DC. | 50 | 0 | 0 | 0 |
|  | *Cortia depressa* (D.Don) C.Norman | 8.33 | 8.33 | 75 | 0 |
| Araceae | *Arisaema utile* Hoo.f. ex Engl. | 33.33 | 0 | 0 | 0 |
| Asteraceae | *Carpesium nepalense* Less. | 0 | 0 | 16.67 | 0 |
|  | *Dubyaea hispida* DC. | 91.67 | 0 | 25 | 0 |
|  | *Jacobea raphanifolia* (Wall. ex Dc.) B. Nord. | 33.33 | 0 | 16.67 | 0 |
|  | *Melanoseris brunoniana* (Wall. ex DC.) N. Kilian & Ze H. Wang | 25 | 0 | 0 | 0 |
|  | *Melanoseris lessertiana* (DC.) Decne | 8.33 | 0 | 0 | 0 |
|  | *Myriactis nepalensis* Lees. | 25 | 0 | 0 | 0 |
|  | *Oreoseris nivea* DC. | 75 | 8.33 | 16.67 | 0 |
|  | *Saussurea eriostemon* Wall. ex C. B. Clarke | 8.33 | 0 | 0 | 0 |
|  | *Saussurea hieracioides* Hook.f. | 16.67 | 0 | 0 | 0 |
|  | *Taraxacum parvulum DC.* | 25 | 16.67 | 41.67 | 0 |
|  | Unidentified sp2 | 75 | 66.67 | 0 | 0 |
| Boraginaceae | *Cynoglossum wallichii* G. Don | 0 | 8.33 | 25 | 0 |
| Campanulaceae | *Cyananthus lobatus* Wall. ex Benth. | 0 | 33.33 | 0 | 0 |
| Caprifoliaceae | *Morina nepalensis* D.Don | 16.67 | 0 | 0 | 0 |
| Caryophyllaceae | *Stellaria* sp. | 41.67 | 0 | 25 | 8.33 |
| Cyperaceae | *Carex nubigena* D.Don | 91.67 | 100 | 58.33 | 83.33 |
|  | *Carex* sp.1 | 83.33 | 0 | 0 | 0 |
|  | *Carex* sp.2 | 0 | 0 | 0 | 16.67 |
|  | *Carex* sp.3 | 0 | 0 | 8.33 | 0 |
| Euphorbiaceae | *Euphorbis wallichii* Hook.f. | 25 | 0 | 25 | 0 |
| Fabaceae | *Medicago edgeworthii*Širj. | 0 | 0 | 16.67 | 0 |
| Gentianaceae | *Halenia elliptica* D.Don | 8.33 | 0 | 0 | 0 |
| Geraniaceae | *Geranium donianum* Sweet | 8.33 | 0 | 0 | 0 |
|  | *Geranium wallichianum* D.Don ex Sweet | 41.67 | 8.33 | 58.33 | 33.33 |
| Hypericaceae | *Hypericum elodeoides* Choisy | 75 | 0 | 0 | 0 |
| Hypodematiaceae | *Hypodematium crenatum* (Forssk.) Kuhn | 8.33 | 0 | 0 | 0 |
| Lamiaceae | *Clinopodium umbrosum* (M.Bieb.) K.Koch | 8.33 | 0 | 8.33 | 0 |
|  | *Mentha longifolia* (L.) L. | 0 | 0 | 16.67 | 0 |
|  | *Nepeta laevigata* (D.Don) Hand.-Mazz. | 66.67 | 0 | 0 | 0 |
|  | Unidentified sp.4 | 0 | 0 | 16. 67 | 0 |
| Onagraceae | *Epilobium brevifolium* D.Don | 66.67 | 58.33 | 8.33 | 0 |
| Orchidaceae | *Herminium josephi* Rchb.f. | 0 | 0 | 8.33 | 0 |
| Orobanchaceae | *Pedicularis gracilis* Wall. ex Benth. | 8.33 | 0 | 0 | 0 |
| Papaveraceae | *Corydalis calycina* Liden | 8.33 | 0 | 0 | 0 |
|  | Unidentified sp.3 | 0 | 0 | 16.67 | 0 |
| Plantaginaceae | *Hemiphragma heterophyllum* Wall. | 66.67 | 0 | 0 | 0 |
|  | *Plantago depressa* Willd. | 8.33 | 8.33 | 8.33 | 8.33 |
| Poaceae | *Agrostis pilosula* Trin. | 50 | 0 | 0 | 0 |
|  | *Poa annua* L. | 41.67 | 83.33 | 100 | 50 |
|  | *Poa rajbhandarii* Noltie | 50 | 91.67 | 50 | 16.67 |
| Polygonaceae | *Bistorta amplexicaulis*(D.Don) Greene | 50 | 8.33 | 41.67 | 16.67 |
|  | *Bistorta macrophylla* (D.Don) Soják | 8.33 | 83.33 | 75 | 0 |
|  | *Koenigia mollis* (D.Don) T.M.Schust. & Reveal | 0 | 25 | 0 | 0 |
|  | *Persicaria nepalensis* (Meisn.) H. Gross | 25 | 58.33 | 41.67 | 8.33 |
|  | *Koenigia mollis* (D.Don) T.M.Schust. & Reveal | 91.67 | 75 | 41.67 | 100 |
|  | *Rumex nepalensis* Spreng. | 41.67 | 75 | 100 | 83.33 |
| Ranunculaceae | *Anemonastrum obtusilobum* (D. Don) Mosyakin | 66.67 | 91.67 | 66.67 | 33.33 |
|  | *Eriocapitella rivularis* (Buch.-Ham. ex DC.) Christenh. & Byng | 33.33 | 16.67 | 0 | 0 |
|  | *Ranunculus brotherusii* Freyn | 16.67 | 25 | 41. 67 | 0 |
|  | *Ranunculus diffusus* DC. | 58.33 | 25 | 41.67 | 8.33 |
| Rosaceae | *Astragalus* sp. | 75 | 0 | 0 | 0 |
|  | *Fragaria nubicola* (Lindl. ex Hook.f.) Lacaita | 41.67 | 8.33 | 41.67 | 8.33 |
|  | *Maharanga bicolor*(Wall. ex G.Don) DC. | 0 | 0 | 8.33 | 0 |
|  | *Potentilla griffithii* Hook.f. | 100 | 8.33 | 16.67 | 41.67 |
|  | *Potentilla lineata* Trevir. | 75 | 0 | 0 | 8.33 |
|  | *Sibbaldia cuneata* Edgew. | 33.33 | 0 | 0 | 0 |
| Saxifragaceae | *Saxifraga parnassifolia* D.Don | 8.33 | 0 | 0 | 0 |
| Violaceae | *Viola biflora* L. | 8.33 | 8.33 | 16.67 | 0 |
| **Khiriya** | | | | | |
| Apiaceae | *Bupleurum longicaule* Wall. ex DC. | 0 | 25 | 25 | 16.67 |
|  | *Ligusticopsis wallichiana* (DC.) Pimenov & Kljuykov | 16.67 | 0 | 0 | 0 |
| Asparagaceae | *Polygonatum singalilense* H. Hara | 8.33 | 0 | 0 | 0 |
| Asteraceae | *Anaphalis contorta* (D. Don) Hook.f. | 58.33 | 25 | 50 | 16.67 |
|  | *Anaphalis nepalensis* (Spreng.) Hand.-Mazz. | 25 | 0 | 0 | 0 |
|  | *Artemisia indica*Willd. | 0 | 0 | 8.33 | 0 |
|  | *Aster diplostephioides*(DC.) C. B. Clarke | 0 | 0 | 8.33 | 0 |
|  | *Cremanthodium arnicoides* (DC. ex Royle) R.D.Good | 16.67 | 0 | 0 | 0 |
|  | *Dolomiaea macrocephala* DC. ex Royle | 0 | 41.67 | 50 | 0 |
|  | *Dubyaea hispida* DC. | 8.33 | 0 | 0 | 0 |
|  | *Hippolytia longifolia* (Rech.f.) C.Shih | 66. 67 | 16.667 | 0 | 8.33 |
|  | *Jacobea raphanifolia* (Wall. ex Dc.) B. Nord. | 33.33 | 0 | 0 | 0 |
|  | *Leontopodium himalayanum* DC. | 25 | 100 | 8.33 | 0 |
|  | *Oreoseris nivea* DC. | 25 | 50 | 66.67 | 33.33 |
|  | *Taraxacum parvulum* DC. | 0 | 0 | 33.33 | 0 |
|  | Unidentified sp.1 | 8.33 | 0 | 0 | 0 |
| Betulaceae | *Betula utilis* D.Don | 8.33 | 0 | 0 | 0 |
| Boraginaceae | *Maharanga bicolor*(Wall. ex G.Don) DC. | 0 | 0 | 8.33 | 0 |
| Campanulaceae | *Cyananthus hookeri* C.B.Clarke | 8.33 | 83.33 | 58.33 | 0 |
|  | *Cyananthus lobatus* Wall. ex Benth. | 8.33 | 0 | 0 | 0 |
| Caprifoliaceae | *Morina nepalensis* D.Don | 0 | 16.67 | 0 | 0 |
| Caryophyllaceae | *Cerastium* sp. | 0 | 16.67 | 8.33 | 0 |
| Cyperaceae | *Carex nubigena* D.Don | 0 | 8.33 | 8.33 | 0 |
|  | *Carex unciniiformis* Boeckeler | 0 | 75 | 41.67 | 0 |
| Dryopteridaceae | *Polystichum bakerianum* (Ark. ex C.B. Clarke) Diels | 16.67 | 0 | 0 | 0 |
| Ericaceae | *Rhododendron anthopogon* D.Don | 16.67 | 0 | 0 | 0 |
| Euphorbiaceae | *Euphorbia stracheyi* Boiss. | 0 | 25 | 8.33 | 0 |
| Fabaceae | *Thermopsis lanceolata* R. Br. ex W.T. Aiton | 0 | 0 | 0 | 16.67 |
| Gentianaceae | *Gentiana* sp. | 0 | 16.67 | 0 | 0 |
|  | *Halenia elliptica* D.Don | 58.33 | 0 | 0 | 0 |
|  | *Swertia angustifolia* Buch. Ham. ex D. Don | 8.33 | 8.33 | 0 | 41.67 |
| Geraniaceae | *Geranium wallichianum* D.Don ex Sweet | 66.67 | 8.33 | 16.67 | 16.67 |
| Iridaceae | *Iris decora* Wall. | 0 | 25 | 75 | 41.67 |
| Lamiaceae | *Prunella vulgaris* L. | 0 | 0 | 8.33 | 0 |
|  | *Salvia campanulata* Wall. ex Benth. | 8.33 | 0 | 0 | 0 |
|  | *Stachys splendens*Wall. ex Benth. | 8.33 | 0 | 0 | 0 |
| Onagraceae | *Epilobium brevifolium* D.Don | 8.33 | 8.33 | 0 | 0 |
| Orchidaceae | *Herminium josephi*Rchb.f. | 0 | 8.33 | 0 | 0 |
| Orobanchaceae | *Pedicularis gracilis* Wall. ex Benth. | 25 | 58.33 | 83.33 | 75 |
|  | *Pedicularis hoffmeisteri* Klotzsch | 16.67 | 0 | 0 | 0 |
| Plantaginaceae | *Veronica himalensis* D. Don | 16.67 | 50 | 25 | 41.67 |
| Poaceae | *Agrostis pilosula* Trin. | 0 | 0 | 25 | 0 |
|  | *Poa annua* L. | 0 | 0 | 8.33 | 0 |
|  | *Tenaxia cumminsii* (Hook.f.) N.P.Barker & H.P.Linder | 41.67 | 83.33 | 91.67 | 100 |
| Polygonaceae | *Bistorta affinis* (D.Don) Greene | 0 | 8.33 | 0 | 0 |
|  | *Bistorta amplexicaulis* (D.Don) Greene | 16.67 | 0 | 0 | 0 |
|  | *Bistorta macrophylla* (D.Don) Soják | 0 | 50 | 8.33 | 66.67 |
|  | *Persicaria nepalensis* (Meisn.) H. Gross | 41.67 | 58.33 | 58.33 | 66.67 |
|  | *Koenigia mollis* (D.Don) T.M.Schust. & Reveal | 66.67 | 0 | 0 | 8.33 |
|  | *Rumex nepalensis* Spreng. | 0 | 8.33 | 25 | 0 |
| Primulaceae | *Primula denticulata* Sm. | 8.33 | 8.33 | 0 | 0 |
| Ranunculaceae | *Anemonastrum polyanthes* (D.Don) Holub | 41.67 | 83.33 | 75 | 100 |
|  | *Delphinium himalayae* Munz | 33.33 | 0 | 0 | 8. 333 |
|  | *Ranunculus brotherusii* Freyn | 0 | 0 | 25 | 0 |
| Rosaceae | *Astragalus* sp. | 0 | 0 | 0 | 41.67 |
|  | *Geum elatum* Wall. f. rubrum Ludlow | 66.67 | 16.67 | 8.33 | 33.33 |
|  | *Potentilla griffithii* Hook.f. | 75 | 66.67 | 66.67 | 83.33 |
|  | *Potentilla lineata*  Trevir. | 0 | 58.33 | 100 | 33.33 |
|  | *Sanguisorba diandra* (Hook.f.) Nordborg | 25 | 0 | 0 | 0 |
|  | *Sibbaldia cuneata* Edgew. | 8.33 | 41.67 | 0 | 41.67 |
| Saxifragaceae | *Saxifraga brachypoda* D. Don | 0 | 8.33 | 0 | 0 |
|  | *Saxifraga parnassifolia*D.Don | 8.33 | 66.67 | 100 | 33.33 |
| Zingiberaceae | *Roscoea*sp. | 0 | 0 | 16.67 | 0 |
