# Supplementary material for: Grazing Intensity Shapes Vegetation Structure and Soil Characteristics in High‐Elevation Rangelands of Nepal
Source: Ecol Evol. 2025 Dec 17;15(12):e72689. doi: 10.1002/ece3.72689 (PMC12711437; doi:10.1002/ece3.72689)
Supplement: Supplementary file 2 — Table S2: Summary table showing (1) stress values from NMDS, (2) PERMANOVA results. [file ECE3-15-e72689-s001.zip › Supplementary table S2.docx]

**Supplementary Table S2: Summary table showing (1) stress values from NMDS, (2) PERMANOVA results**

| **Variables** | **Group** | **Value** | **R^2^** | **P value** |
| --- | --- | --- | --- | --- |
| NMDS stress | Dargari | 0.117 | NA | 0.001 |
| NMDS stress | Khali | 0.188 | NA | 0.001 |
| NMDS stress | Khiriya | 0.19 | NA | 0.001 |
| PERMANOVA | Sites | 29.394 | 0.29 | 0.001 |
| PERMANOVA | Grazing intensity | 8.224 | 0.15 | 0.001 |
| PERMANOVA | Sites × Grazing intensity | 27.577 | 0.69 | 0.001 |
| **Posthoc test** | | | | |
| **Dargari** | LG*MG | 24.07167 | 0.52 | 0.001 |
|  | LG*NG | 43.6566 | 0.66 | 0.001 |
|  | LG*HG | 29.98 | 0.58 | 0.001 |
|  | MG*NG | 39.47 | 0.64 | 0.001 |
|  | MG*HG | 39.09 | 0.64 | 0.001 |
|  | NG*HG | 36.23 | 0.62 | 0.001 |
| **Khali** | LG*MG | 6.73 | 0.23 | 0.001 |
|  | LG*NG | 22.27 | 0.5 | 0.001 |
|  | LG*HG | 14.78 | 0.4 | 0.001 |
|  | MG*NG | 15.21 | 0.41 | 0.001 |
|  | MG*HG | 11.63 | 0.35 | 0.001 |
|  | NG*HG | 22.65 | 0.51 | 0.001 |
| **Khiriya** | LG*MG | 5.58 | 0.2 | 0.001 |
|  | LG*NG | 13.68 | 0.38 | 0.001 |
|  | LG*HG | 8.35 | 0.28 | 0.001 |
|  | MG*NG | 14.68 | 0.4 | 0.001 |
|  | MG*HG | 8.67 | 0.28 | 0.001 |
|  | NG*HG | 10.39 | 0.32 | 0.001 |
